# Understanding LGBTQ health in women’s health nursing: definitions, lifespan health needs, and inclusive care

**DOI:** 10.4069/whn.2026.03.17

**Published:** 2026-03-31

**Authors:** Sook Jung Kang

**Affiliations:** College of Nursing, Ewha Womans University, Seoul, Korea

## Introduction

The health of lesbian, gay, bisexual, transgender, and queer or questioning (LGBTQ) populations is a fundamental component of women’s health nursing, rather than a marginal concern. In gynecological, reproductive health, and preventive care settings, nurses should use non-assumptive, person-centered assessment that addresses pertinent sexual and reproductive health needs, anatomy-based screening, and communication that protects privacy [1-3]. This is important because women’s health nursing encompasses care for individuals whose sexual orientation, gender identity, gender expression, anatomy, and family structures do not conform to heterosexual or cisgender norms. When clinicians presume heterosexuality, cisgender identity, or a direct correspondence between gender identity and anatomy, assessments may lack precision and care may be perceived as unsafe or disrespectful. Such presumptions can constrain discussions of sexually transmitted infection (STI) prevention, cervical cancer screening, fertility counseling, and routine gynecological care. Importantly, the social context surrounding LGBTQ identification—including stigma, discrimination, and the risk of misidentification in healthcare settings—has a greater influence on health outcomes than identity alone [4-7].

## Key terminology

Sex assigned at birth refers to the classification recorded at birth based on physical characteristics. Gender identity refers to an individual’s internal sense of gender, including how they understand their gender and how they wish to be recognized by others. Sexual orientation is defined as a persistent pattern of romantic, emotional, or sexual attraction, including the genders toward which such attraction is directed [8]. LGBTQ is an umbrella term encompassing lesbian, gay, bisexual, transgender, queer or questioning, and other identities outside dominant sexual and gender norms, although the terminology used to describe these groups continues to evolve [9]. Sexual orientation and gender identity (SOGI) is a clinically useful construct because it enables clinicians to avoid assumptions, ask more relevant questions, and identify disparities in access to preventive services. Recent work on SOGI data collection has emphasized the use of standardized questions, the two-step approach (assessing current gender identity and sex assigned at birth), protection of privacy, and implementation strategies that align with clinical workflows [10-14].

## Health equity perspective

Health inequalities affecting LGBTQ populations are more appropriately attributed to stigma, discrimination, exclusion, and inequitable access to affirming care than to identity itself. A health equity perspective emphasizes preventable and unjust disparities, shifting the focus from individual characteristics to the social and clinical structures that shape care [4,15]. Minority stress theory explains how chronic stress arising from discrimination, anticipated rejection, concealment, and repeated invalidation accumulates over time and influences mental health [5]. These processes operate not only through interpersonal interactions but also through structural stigma, such as registration systems that enforce inaccurate categorization and institutional practices that fail to recognize partners or chosen family [7]. Evidence from Korea similarly indicates that discrimination is associated with delayed or avoided healthcare among transgender adults [6]. In women’s health nursing, this perspective is particularly relevant because many clinical encounters involve sensitive issues related to sexuality, reproduction, and family dynamics.

## Why LGBTQ health matters in women’s health nursing

Knowledge of LGBTQ health is essential in women’s health nursing for three primary reasons. First, many routine assessments depend on anatomy, sexual practices, and partnership patterns rather than assumptions about identity. Second, patients may delay or avoid care due to fear of criticism or misunderstanding, which may adversely affect screening, follow-up, and prevention. Third, communication itself functions as a clinical intervention. Inclusive women’s health nursing therefore involves explaining the purpose of SOGI questions, allowing patients to decline, and minimizing unnecessary disclosure [1,3,10-14]. These principles apply to sexual history taking, counseling, the use of names and pronouns, and clinical documentation in routine practice. Screening decisions should be guided by anatomy and clinical history rather than identity labels alone [16]. This approach is particularly important when addressing pregnancy intentions, STI prevention, fertility counseling, and cervical cancer screening. Care for transgender and gender-diverse patients similarly requires attention to preventive and sexual health needs that may not be evident from identity labels alone [17]. This educational need is also reflected in nursing curricula. In one report, faculty at two U.S. schools of nursing described the development of stand-alone LGBTQIA+ (lesbian, gay, bisexual, transgender, queer or questioning, intersex, and asexual) health electives after identifying gaps in existing curricula, suggesting that structured inclusion of LGBTQ health content is both feasible and educationally meaningful [18].

## Lifespan health needs

LGBTQ health needs vary across the life course [7]. During adolescence and emerging adulthood, key concerns include identity development, family conflict, exposure to violence, depression, anxiety, suicidality, and access to STI prevention and contraception without heteronormative assumptions [19]. Young people may also require confidential counseling and trauma-informed communication, particularly when disclosure at home or school poses a risk. In adulthood and midlife, important issues include chronic stress, preventive screening, reproductive counseling, workplace and caregiving responsibilities, and the need for care based on relevant anatomy and clinical history [16]. Preventive services may be underutilized when clinicians equate gender identity with anatomy or assume heterosexual partnership patterns. In older adulthood, major concerns include social isolation, limited support networks, discrimination in long-term care, cognitive decline, multimorbidity, and challenges related to surrogate decision-making [20]. Women’s health nurses should therefore consider age and social context jointly rather than treating LGBTQ populations as a homogeneous group.

## Implications for inclusive care

Inclusive women’s health nursing begins with non-assumptive communication. Nurses should ask relevant questions as a routine component of care, explain that these questions are intended to support safety and accurate assessment, and allow patients to decline [1]. Clinicians should inquire about partners, sexual activity, and reproductive concerns without presuming gender, identity, or family structure. Decisions regarding screening and counseling should be informed by relevant anatomy and clinical history rather than identity-based assumptions alone [12,16]. Respectful use of names and pronouns, protection of privacy, and careful documentation are equally important, as they influence whether patients feel safe returning for care [3,11,13,14,21]. Attention must also be given to the risk of unintended disclosure in waiting areas, electronic records, and conversations with family members. In practice, inclusive care is achieved through neutral opening questions, accurate documentation language, clarification of who should be involved in communication and decision-making, and recognition of chosen family when appropriate [3,7].

## Implications for education and policy

These clinical principles require support from nursing education and institutional policy. Women’s health nursing curricula should include foundational content on terminology, SOGI-informed assessment, anatomy-based screening, and confidentiality [2,3,21]. Education should emphasize that LGBTQ health is not a specialized topic separate from routine practice, but an integral component of competent assessment, communication, and prevention across the life course. Health systems should implement documentation practices, workflows, and staff training that promote respectful communication and equitable access to preventive services [10,13,14]. In the Korean context, broader integration of LGBTQ health into nursing education and practice standards may reduce uncertainty, enhance consistency, and support more accountable care. Such efforts may improve nurses’ confidence in providing care for LGBTQ patients and strengthen their capacity to deliver respectful, competent, and responsive care in clinical settings.

## Conclusion

Providing inclusive care for sexual and gender minority populations is increasingly recognized as a central responsibility in women’s health nursing across the lifespan. Key priorities include the use of precise language, adoption of a health equity perspective, recognition of minority stress, and routine communication that avoids presumptions while protecting patient privacy. Strengthening these conceptual foundations within women’s health nursing education and practice may promote safer, more reflective, and more equitable care.
